# Maintaining neglected tropical disease programmes during pandemics

**DOI:** 10.2471/BLT.20.269464

**Published:** 2021-04-01

**Authors:** Jared M Alswang, Alexis L Gutierrez, Samantha J Sadler, Ole F Norheim

**Affiliations:** a Harvard Medical School, 25 Shattuck St, Boston, MA 02115, United States of America.; b Department of Global Health and Primary Care, University of Bergen, Bergen, Norway.

Neglected tropical diseases comprise 20 communicable diseases of different pathogenic origins endemic to tropical and subtropical regions. These diseases disproportionately affect the world’s poorest and most marginalized populations.^1^ The global burden of neglected tropical diseases is devastating, as it affects the lives and economies of over 1.6 billion people.^2^

Fortunately, in the last decade, neglected tropical diseases have begun receiving global public health attention. Two movements prioritizing treatment for such diseases were launched in 2012: the London Declaration on Neglected Tropical Diseases and the World Health Organization’s (WHO) *Accelerating work to overcome the global impact of neglected tropical diseases: a roadmap for implementation*.^2^ These two international agreements have helped shift the global perception of such diseases from irremediable casualties of poverty to unacceptable manifestations of global inequalities. The World Bank’s *Disease control priorities in developing countries* identifies mass drug administration, vector management and early detection and treatment programmes as having high economic impact, and therefore as priority approaches to attenuating neglected tropical disease burden in low-resource settings.^3^

The signatories of the London Declaration on Neglected Tropical Diseases have made substantial progress towards reaching the ambitious targets of neglected tropical disease management outlined in 2012. In 2018 alone, 1.12 billion people received treatment for these diseases and 1.75 billion individuals at risk were identified through mapping efforts.^1^ These efforts have translated into a reduction in caseloads, with some nations even achieving the elimination of certain neglected tropical diseases, therefore decreasing the burden of disease on vulnerable populations worldwide.^2^

However, changes in the geopolitical climate and fluctuations in funding frequently threaten to derail the momentum. Unexpected shifts in health priorities leave neglected tropical diseases vulnerable to once again becoming neglected. A particularly salient example of this occurred during the 2013–2016 Ebola virus disease outbreak in West Africa, demonstrating how emerging diseases threaten the substantial progress made towards eliminating neglected tropical diseases.

## Ebola virus disease

In 2014, the Ebola virus disease epidemic sparked unprecedented media coverage around the world. The high case fatality rate, in conjunction with the symptoms of the virus, fed a media-driven sense of urgency that turned the attention of the world to West Africa. The outbreak hit Guinea, Liberia and Sierra Leone the hardest, all of which were already substantially burdened by neglected tropical diseases.^4^ Given the relatively low mortality, yet high morbidity of neglected tropical diseases, the Ebola virus outbreak impeded the mitigation efforts of these diseases.

As international attention and global health efforts shifted to Ebola virus disease containment, neglected tropical disease programmes, which took years to effectively implement, were halted in the areas where Ebola virus disease was most prevalent.^5^ Expansion of endemic neglected tropical disease treatment and prevention hinges on few resources that are delicate and difficult to establish, namely: trust in the health system, efficient transport of medications and supplies and trained health-care personnel.^5^^,^^6^ With this outwardly more pressing outbreak occurring, nearly all existing health resources and infrastructure were reallocated to combat the spread of Ebola. The actions taken to combat the outbreak in these countries undoubtedly saved lives, but the prioritization of Ebola virus disease came at the detriment of endemic disease management.

The concurrent burden of disease from neglected tropical disease is an important consideration during outbreaks. A study estimates that almost half of the total population of the three countries hardest hit by the outbreak has at least one neglected tropical disease and/or malaria.^4^ Aware of the threat to existing health programmes that the outbreak posed, WHO released guidelines for temporary recommendations to allocate resources to continue to combat malaria. WHO based its reasoning on the notion that fewer hospital admissions from diseases other than Ebola would limit the spread of the virus within the health-care system and minimize the volume of patients, allowing more resources for those infected with the Ebola virus.^7^ However, no explicit recommendations were made to continue neglected tropical disease prevention programmes during the outbreak.^4^ As a result, many national neglected tropical disease programmes froze operations for up to two full years.^5^

Once the Ebola outbreak appeared adequately contained, neglected tropical diseases and other public health programmes were permitted to resume their respective programming. However, the partial to complete cessation of these programmes during the outbreak reintroduced, and in some cases worsened, past implementation challenges of treatment and prevention programmes for neglected tropical diseases.^5^ Notable challenges included heightened mistrust of health workers, reestablishment of logistics and retraining of staff.^5^^,^^6^ The fragile social and political structures for integrated mass drug administration programmes needed to be rebuilt, prolonging and compounding the suffering as a result of untreated neglected tropical diseases in the interim.

Ultimately, the temporary yet dramatic displacement of health resources during the outbreak not only permitted regression in neglected tropical disease control, but may have also exacerbated the overall burden on already resource-constrained health-care systems during the epidemic.^4^^,^^6^

## Epidemic to pandemic

Holistic guidelines to adapt and maintain neglected tropical disease treatment and prevention are needed to prepare for the next inevitable epidemic – or pandemic. However, historically, efforts to synthesize guidelines from best practices during specific epidemic (or pandemic) responses have been inadequate. A recent study found the methodological and reporting quality of general governing guidelines for the severe acute respiratory syndrome coronavirus 2 (SARS-CoV-2) pandemic are insufficient to manage the public health crisis; the same happened during the Ebola virus disease, Zika virus and other outbreaks.^8^

Programme management for neglected tropical disease is no exception. For example, in June 2020, WHO advised that community-based neglected tropical disease interventions, including mass treatment, community-based surveys and active case-finding, be postponed until further notice in the wake of the SARS-CoV-2 outbreak.^9^ The decision to pause these programmes also contrasts with WHO’s recommendations in 2017 following the Ebola virus disease outbreak, where maintenance of neglected tropical disease treatment, surveillance and multidisciplinary programmes was described as critical to disease management.^10^

These conflicting recommendations highlight both the inefficiency with which we have tackled epidemics in the past and the lack of progress since. The health community should recognize and learn from these shortcomings during the current pandemic, for both present and future practices. With the heightened propensity for pandemics to affect operations that are not directly related to their management, neglected tropical disease management is under an even greater threat than before. Therefore, having robust guidelines on securing and efficiently using programme resources and infrastructure is critical to optimize neglected tropical disease operations during pandemics.

## Local vs global

As our knowledge of SARS-CoV-2 continues to evolve, so does our understanding of fundamental practices key to containing the virus. Importantly, differences in context and culture between affected communities often inform their respective approaches to implementing infection control practices.^11^ Local stakeholders should be involved when determining the most effective interventions for a given local setting,^12^ including how to appropriately tailor resource allocation for neglected tropical diseases as part of the larger conversation around SARS-CoV-2. International guidelines for management of such diseases in pandemic settings should therefore include frameworks for effective local adaptation of core principles and recommendations. Moreover, prioritizing locally informed approaches will help secure the involvement of local stakeholders, reinforcing the care for people with neglected tropical diseases.

Historical precedent and increasing globalization make us predict that SARS-CoV-2 will not be the last pandemic to uproot global health infrastructure. Given the progress made thus far in mitigating the disproportionate neglected tropical disease burden on low- and middle-income countries, efforts must be made for resource allocation to allow evidence-based programmes to be prioritized and not compromised in the wake of other pandemic responses.

The regression of neglected tropical disease management seen during the Ebola outbreak foreshadows what may happen on a global scale if we fail to learn from past outbreaks. As the SARS-CoV-2 pandemic continues to unfold, studies are needed to: (i) clarify why and how neglected tropical disease resources shift; (ii) understand the impact of these resource shifts on neglected tropical disease burden; and (iii) understand the perspectives of local stakeholders who control and/or receive these resources in preparation for inevitable future pandemics. Moreover, integrating these findings with lessons learnt from current practice is critical to inform unified guidelines for optimal resource allocation to neglected tropical disease management during novel disease outbreaks. An improved understanding of how management of these diseases might be best leveraged during a pandemic to optimize resources and minimize loss of life is imperative to overcoming the current SARS-CoV-2 pandemic and informing our approach to the next outbreak.

## References

[1] Engels D, Zhou X-N. Neglected tropical diseases: an effective global response to local poverty-related disease priorities. Infect Dis Poverty. 2020 1 28;9(1):10. 10.1186/s40249-020-0630-9 31987053 PMC6986060

[2] The Lancet Global Health. Taking the neglected out of neglected tropical diseases. Lancet Glob Health. 2020 2;8(2):e152. 10.1016/S2214-109X(19)30529-7 31981542

[3] Jamison DT, Alwan A, Mock CN, Nugent R, Watkins D, Adeyi O, et al. Universal health coverage and intersectoral action for health: key messages from Disease Control Priorities, 3rd edition. Lancet. 2018 3 17;391(10125):1108–20. 10.1016/S0140-6736(17)32906-9 29179954 PMC5996988

[4] Hotez PJ. Neglected tropical diseases in the Ebola-affected countries of West Africa. PLoS Negl Trop Dis. 2015 6 25;9(6):e0003671. 10.1371/journal.pntd.0003671 26110778 PMC4482537

[5] Bogus J, Gankpala L, Fischer K, Krentel A, Weil GJ, Fischer PU, et al. Community attitudes toward mass drug administration for control and elimination of neglected tropical diseases after the 2014 outbreak of Ebola virus disease in Lofa county, Liberia. Am J Trop Med Hyg. 2016 3;94(3):497–503. 10.4269/ajtmh.15-0591 26666700 PMC4775880

[6] Thomas BC, Kollie K, Koudou B, Mackenzie C. Commentary: restarting NTD programme activities after the Ebola outbreak in Liberia. Infect Dis Poverty. 2017 5 1;6(1):52. 10.1186/s40249-017-0272-8 28457226 PMC5410695

[7] Guidance on temporary malaria control measures in Ebola-affected countries. Geneva: World Health Organization; 2014. Available from: https://apps.who.int/iris/handle/10665/141493?show=full [cited 2020 May 6].

[8] Zhao S, Cao J, Shi Q, Wang Z, Estill J, Lu S, et al.; COVID-19 evidence and recommendations working group. A quality evaluation of guidelines on five different viruses causing public health emergencies of international concern. Ann Transl Med. 2020 4;8(7):500. 10.21037/atm.2020.03.130 32395544 PMC7210117

[9] Maintaining essential health services. Operational guidance for the COVID-19 context: interim guidance, 1 June 2020. Geneva: World Health Organization; 2020. Available from: https://apps.who.int/iris/handle/10665/332240 [cited 2020 Jun 5].

[10] Integrating neglected tropical diseases into global health and development. Fourth WHO report on neglected tropical diseases. Geneva: World Health Organization; 2017. Available from: https://apps.who.int/iris/handle/10665/255011 [cited 2020 May 25].

[11] Khanna RC, Cicinelli MV, Gilbert SS, Honavar SG, Murthy GSV. COVID-19 pandemic: lessons learned and future directions. Indian J Ophthalmol. 2020 5;68(5):703–10. 10.4103/ijo.IJO_843_20 32317432 PMC7350475

[12] Molyneux DH, Aboe A, Isiyaku S, Bush S. COVID-19 and neglected tropical diseases in Africa: impacts, interactions, consequences. Int Health. 2020 9 1;12(5):367–72. 10.1093/inthealth/ihaa040 32725145 PMC7443717

